# Mandibular Regional Odontodysplasia in an 8-year-old Boy showing Teeth Disorders, Gubernaculum Tracts, and Altered Bone Fractal Pattern

**DOI:** 10.5005/jp-journals-10005-1498

**Published:** 2018-04-01

**Authors:** Davi de Sá Cavalcante, Cristiane SR Fonteles, Thyciana R Ribeiro, Lúcio M Kurita, Alynne Vde M Pimenta, Francisco SR Carvalho, Fábio WG Costa

**Affiliations:** 1 Postgraduate Student, Division of Oral Radiology, Federal University of Ceará Fortaleza, Ceará, Brazil; 2 Associate Professor, Division of Pediatric Dentistry, Federal University of Ceará Fortaleza, Ceará, Brazil; 3 Adjunct Professor, Division of Special Needs in Dentistry, Federal University of Ceará, Fortaleza, Ceará, Brazil; 4 Adjunct Professor, Division of Oral Radiology, Federal University of Ceará Fortaleza, Ceará, Brazil; 5 Adjunct Professor, Division of Oral Radiology, Federal University of Ceará Fortaleza, Ceará, Brazil; 6 Postgraduate Student, Postgraduate Program in Dentistry, Federal University of Ceará Fortaleza, Ceará, Brazil; 7 Adjunct Professor, Division of Oral Radiology, Federal University of Ceará Fortaleza, Ceará, Brazil

**Keywords:** Cone beam computed tomography, Fractal analysis, Gubernaculum tracts, Mandible, Regional odonto-dysplasia.

## Abstract

Regional odontodysplasia (RO) is a rare nonhereditary developmental condition usually affecting maxilla and female patients. A few number of articles have used the cone beam computed tomography (CBCT) to describe imaging findings in RO. Thus, this article aims to report unusual imaging findings in a Brazilian child who presented with mandibular RO. A healthy 8-year-old boy was referred for evaluation of an anterior mandibular gingival enlargement. Radiographically, all right mandibular teeth showed wide pulp chambers and shortened roots with thin dentinal walls and open apices, featuring a “ghost teeth“ appearance, which indicated a diagnosis of RO. Missing teeth and altered radiopacity were observed. Cone beam computed tomography provided great insight into RO, allowing to identify gubernaculum tracts (GTs) associated with RO teeth. Fractal analysis of the bone surrounding RO teeth revealed an altered trabecular pattern.

**Clinical significance:**

This case describes a rare developmental teeth-related pathology in a boy who showed unusual features on imaging exams. The CBCT provided the observation of RO tooth-related GTs, probably a new finding added to the international literature regarding RO, as well as the pulp chamber volume characterization of an affected tooth not published yet. In addition, it was observed an altered fractal pattern of the mandibular bone adjacent to RO teeth, which has not been described to date.

**How to cite this article:** de Sa Cavalcante D, Fonteles CSR, Ribeiro TR, Kurita LM, Pimenta AVM, Carvalho FSR, Costa FWG. Mandibular Regional Odontodysplasia in an 8-year-old Boy showing Teeth Disorders, Gubernaculum Tracts, and Altered Bone Fractal Pattern. Int J Clin Pediatr Dent 2018;11(2):128-134.

## INTRODUCTION

Tooth anomalies encompass a heterogeneous group of dental disorders related to genetic, epigenetic, and environmental factor interaction during the morpho- or histodifferentiation stages of tooth development.^1^ General tooth dysplasia is a dental condition associated with specific disorders of root development, including double teeth, dentin dysplasia type I, hypophosphatasia, and RO.^2^ This latter is a rare nonhereditary developmental condition usually localized, although it can be generalized or associated with a syndrome that exhibits clinical, and characteristic imaging features.^3^ Although teeth alterations without other extraoral findings are the usual clinical presentation of RO, it has been reported RO teeth simultaneously with epidermal nevus syndrome,^4,5^ hypo-phosphatasia,^6^ hydrocephalus,^7^ and Hallermann-Streiff syndrome.^8^ These associations with systemic conditions reinforce the uncertain etiology of the RO, which has been related to other factors, such as a viral infection,^9^ use of inappropriate medication during pregnancy,^10^ trauma, nutritional deficiency, infection, and metabolic abnormalities.^11^

Teeth affected by RO show clinical and radiograph aspects useful as diagnostic criteria.^11^ Intraoral inspection may reveal hypoplastic malformed teeth with a yellowish/brownish discoloration.^12^ Plane radiographs are commonly requested for dental evaluation in RO. Panoramic radiograph showed a characteristically unilateral pattern without tendency to cross the midline and a predominant maxillary involvement. Radiographically, the affected teeth have a poor definition between hard dental tissues, as well as aspects resembling an arrested development including large pulp chambers (ghostlike appearance) and short roots with open apices.^13-17^

Imaging exams may also demonstrate delayed eruption of affected teeth, failure in the dental eruption process, tooth agenesis,^18^ or even altered mandibular radioden-sity.^19^ These features are important for the differential diagnosis with hereditary conditions, including dentin dysplasia, dentinogenesis imperfecta, and amelogenesis imperfecta.^20^

Conventional radiographs possess restrictions in terms of imaging details and three-dimensional evaluation. In RO, CT is a useful exam to provide a tri-dimensional tooth morphology, assessment of the pulp chamber volume, tissue density, and localization of dysplasia and hypocal-cification.^21^ However, there is only one published case of RO evaluated on CBCT to date.^8^

Thus, the aim of this paper is to describe teeth imaging findings observed on panoramic radiograph and CBCT from a male patient with mandibular RO, bone fractal properties not published yet, and to add a new feature of this condition.

## CASE REPORT

An 8-year-old boy was referred to the Pediatric Dental Clinic at the Federal University of Ceará (Fortaleza, Ceará, Brazil) for the evaluation of a gingival enlargement of unknown duration situated in the anterior mandibular region associated with tooth pain. The patient showed normal stature and no other physical abnormalities. History of trauma, infectious diseases, or nutritional disorders was absent. Medical abnormalities or dental alterations were not observed among other members of his family. Intraoral examination revealed right mandibular malformed and hypoplastic teeth, deciduous teeth (inferior central incisors) associated with a localized gingival enlargement covered by a fibrous tissue, and teeth 83, 84, and 85 affected by caries. All other teeth were present and normal. At the first visit, clinical data were collected to establish the treatment plan and imaging exams were analyzed. However, after the return visit, the patient did not attend for dental follow-up.

**Fig. 1:**
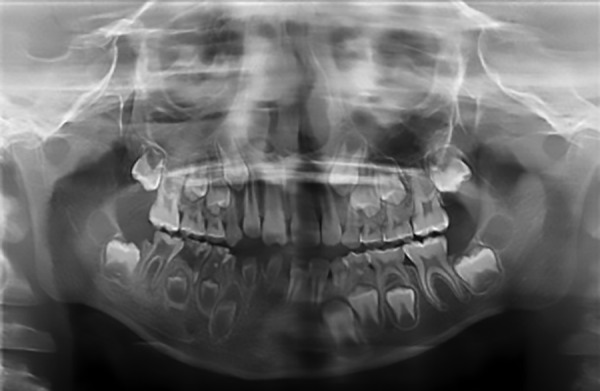
Panoramic radiograph in which deciduous and permanent teeth in the right hemimandibular show large pulp chambers. Agenesis of teeth 41 and 42 is observed, as well as images suggestive of caries on teeth 84, 85, and 46

Panoramic radiograph (Fig. 1) showed primary mandibular teeth (81, 82, 83, 84, and 85), tooth 46 and dental germs (43, 44, and 45) with “ghost teeth” appearance due to the presence of extensive pulp chamber demarcated by thin layer of mineralized tissue in which it was not possible to observe a clear definition of enamel and dentin. In addition, it was revealed agenesis of lower incisor teeth (41 and 42) and images suggesting dental caries on teeth 84, 85, and 46.

**Figs 2A to C:**
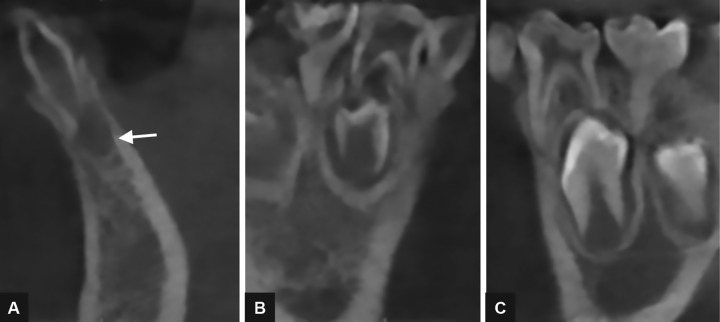
Transverse images of CBCT showing the tooth 82 with a “ghost tooth” aspect associated with a hypodense image (white arrow) suggestive of periapical lesion (A); in addition, a significant difference between the space of the pulp chambers of primary teeth 84 (B) and 74 (C)

**Figs 3A and B:**
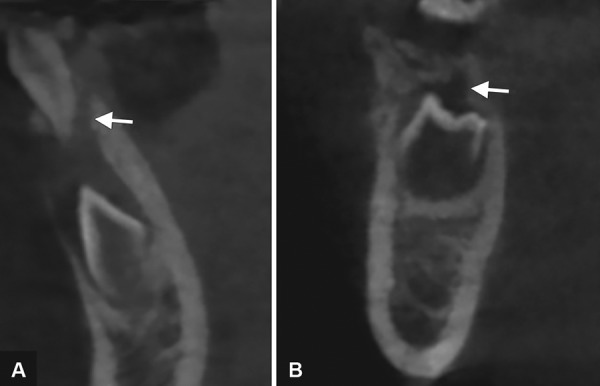
Presence of GTs (white arrows) associated with tooth germs 43 and 45. Also, enlargement of the pericoronal follicle space relative to the tooth germ 43

**Figs 4A to D:**
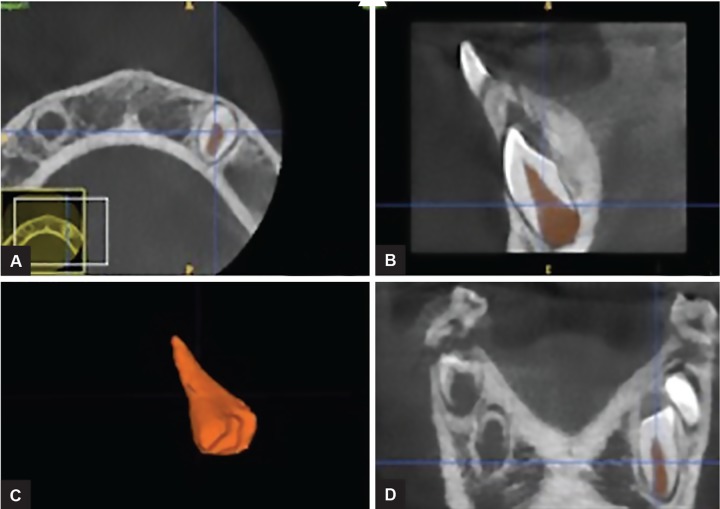
Window of the ITK-SNAP program in which the space of the pulp chamber of the healthy tooth 33 was segmented and rendered, getting a volume of 96.33 mm^3^. Axial, sagittal, three-dimensional, and coronal views are shown

**Figs 5A to D:**
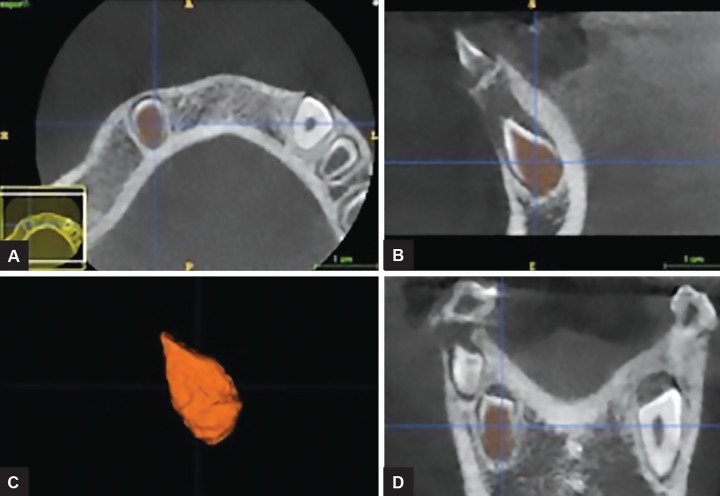
Window of the ITK-SNAP program showing the three-dimensional reconstruction of the space of the pulp chamber of the tooth 43 with OR, which obtained a volume of 128.7 mm^3^. Axial, sagittal, three-dimensional, and coronal views are shown

**Fig. 6:**
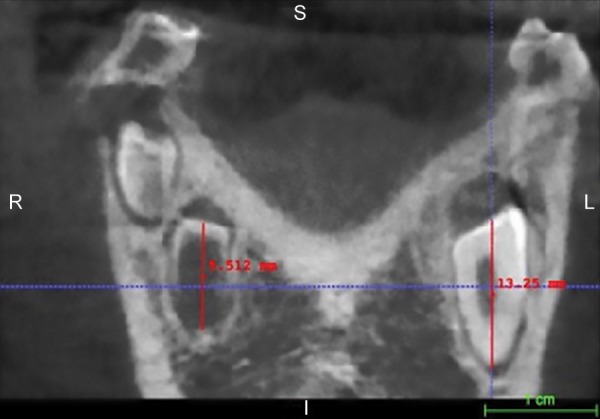
Linear measurements performed in the ITK-SNAP program in which the RO tooth presented a length of 9.512 mm while its contralateral tooth showed a length of 13.25 mm

A CBCT scan revealed: (1) a hypodense area suggestive of a periapical lesion associated with tooth 81, which did not show signs of dental caries (Fig. 2A); (2) remarkable difference in the pulp chamber space when comparing deciduous teeth 84 and 74 (Figs 2B and C); (3) crows of the affected teeth surrounded by large hypodense areas (Fig. 3A); (4) presence of GTs associated with unerupted teeth 43 and 45 (Fig. 3). In order to provide pulp chamber volume, it was used the semi-automated segmentation tool of the free access ITK-SNAP 3.4.0® software (Cognitica, Philadelphia, USA). It was observed that the volume of the RO-positive tooth pulp chamber (Fig. 4) was approximately 25% greater than the volume of the opposite unaffected tooth (Fig. 5). In addition, RO tooth was of approximately 28% shorter length than the corresponding contralateral tooth (Fig. 6).

**Figs 7A to N:**
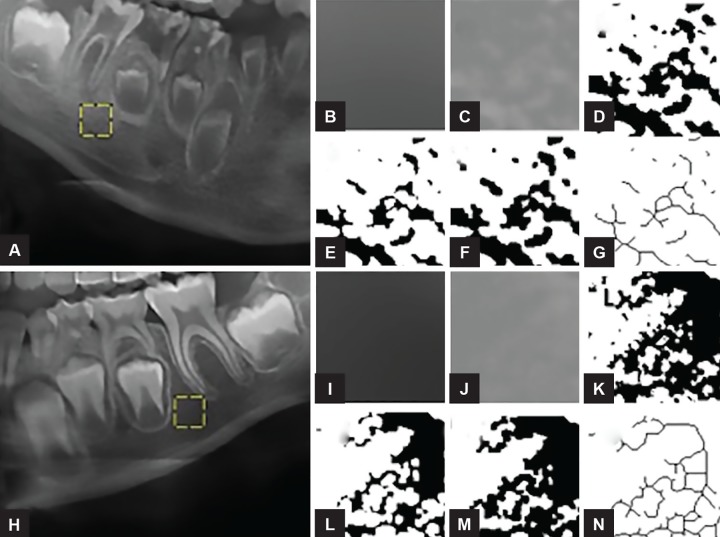
Sequence to obtain the fractal dimension. Regions of interest represented by yellow boxes (A,H) were cropped, duplicated, and a Gaussian filter with a sigma value of 35 (B,I) was applied. Then, the blurred image was subtracted from the original image and 128 was added (C, J). The resulting image was binarized (D, K), eroded (E, L), dilated (F, M) and skeletonized (G, N) in order to calculate the fractal dimension value

Imaging exams also showed an intense radiopacity pattern in the region of the trabecular bone surrounding adjacent RO teeth in comparison with the unaffected con-tralateral region. Thus, a fractal analysis was performed aiming to analyze the mandibular trabecular pattern of both regions (Fig. 7). ImageJ version 1.38x software (US National Institutes of Health, http://rsb.info.nih.gov/ nih-image) was used to perform this analysis. Initially, a region of interest (ROI) of 100 × 100 pixels above the mandibular canal was selected between the mesial roots of the tooth 46 and the unerupted tooth 45. This ROI was similarly selected for both right and left mandibular sides. Then, the procedures for calculating the fractal dimension followed the methodology described by White and Rudolph.^22^ The bone surrounding RO teeth had a fractal dimension value of 1,171, differing from the unaffected bone (value of 1.309), which indicates an altered bone microarchitecture in the mandibular region containing the RO teeth.

## DISCUSSION

The RO is a rare developmental dental anomaly with an estimated prevalence of less than 1:1,000,000, and approximately 140 cases have been published to date.^13,15^ According to Crawford and Aldred,^11^ this condition probably was firstly described in 1934 by Hitchin under the term “arrested tooth development.” Zegarelli et al^23^ introduced the term “odontodysplasia” followed by Pindborg, who designed the term “regional” in order to reflect the clinical finding of involvement in a region or a segment of the affected jaw.^11^ Since its first publication, a few number of Brazilian studies have been performed. A PubMed search of the literature using the keywords “regional odontodysplasia” and “Brazil” disclosed only 10 publications to date, which emphasizes the importance of the present report.

As recorded at the dental appointment for patient evaluating, RO-related teeth are often small, discolored, hypocalcified, and hypoplastic, which reflect their abnormal development regarding dental hard tissues originated from the embryonic layers ectoderm and mesoderm.^16^ In addition, this case is uncommon since RO has been published mainly among females (1.7:1) and with a predilection to maxillary localization (1.6:1).^24^ Presently, it was reported a right mandibular RO in a young boy. According to Babu et al,^20^ there have been only four published cases of RO-positive teeth in mandibular site. In jaws, the third and fourth quadrants have been described as the lower involved regions followed by the first and second quadrants.^24^

In the field of the oral and maxillofacial radiology, imaging findings are strong diagnostic criteria for RO,^11^ typically featuring remarkable alterations in dental tissues of deciduous and/or permanent teeth. This case showed teeth with hypoplastic, hypocalcified crown, poor contrast between dental tissues demonstrated by a low radiodensity, shortened roots with wide open apices, pericoronal radiolucency representing an enlarged dental follicle, and large pulp chambers giving a “ghost teeth” appearance.^11,17,21,23,25,26^ In addition, it was observed the absence of right permanent mandibular central and lateral incisors, an unusual finding according to Tervonen et al.^24^ These authors compiled epidemiological data of RO published in the international literature up to 2002 and found only 10.7% of missing teeth in the studied sample of 138 cases. Similarly to the present case, Ganguly and Ramesh^27^ reported an unusual case of mandibular RO associated with the absence of left lower and lateral incisors which was observed on periapical radiographs.

It has been considered that unknown local factors affecting the tooth-forming tissues during development are probably involved in RO origin.^28^ We believe this fact may explain the occurrence of GTs in RO, which were firstly described in the present report. GT consists of an intraosseous tooth-related eruption pathway containing a fibrous tissue band (gubernacular cord) that communicates the tooth bud follicle with its overlying oral mucosa, appearing in the lingual alveolar bone of the deciduous related tooth.^29-31^ In the present case, CBCT characterized the gubernacular cord-containing pathway as a tooth bud-related hypodense channel as described by Ide et al.^31^ Interestingly, these authors reported a case of a mandibular adenomatoid odontogenic tumor arising in an 11-year-old girl which was associated with a GT on computed tomography. Oda et al^32^ showed a close spatial relationship and/or association between odontomas and the GT on CBCTs, noticeable in exams of individuals less than 20 years of age (>96%). Indeed, benign odontogenic tumors represent a heterogeneous group of lesions developed from the various components of the odontogenic apparatus,^33^ which encompass some similarity with RO regarding etiopathogenesis. In addition, the present case emphasizes the importance of CBCT to accurately detect GT, since panoramic radiography usually does not reveal this finding.^30^

Conventional radiographs usually describe teeth affected by RO with a typical hypoplastic morphology, encompassing large pulp chambers enclosed by thin layers of enamel and dentin.^14^ The digital imaging and communications (DICOM) files originated from CBCT scans and subsequently analyzed by ITK-SNAP software would be more advantageous in order to accurately provide a detailed pulp chamber description as performed in the present case. The use of ITK-SNAP as a valid tool for a tri-dimensional evaluation has been observed in several studies, including volumetric assessment of Stafne’s defects,^28^ forensic anthropology population characterization,^34,35^ monitoring of endodontic treatment outcome in periapical lesions,^36^ and orthodontics purposes.^37,38^ Throughout ITK-SNAP software, a morphological difference between mandibular RO tooth and its nonaffected contralateral tooth was presently evident, since affected tooth showed a pulp chamber volume 25% greater than the normal tooth. Clinically, this finding may predispose to dental fragility, as well as to enhance the occurrence and progression of periapical lesions. In addition, the same tooth evaluated in the present case showed a reduction in length (approximately 28%) when compared with the tooth 33. This aspect may reflect the clinical evidence of a delayed tooth development since RO tooth resembled Nolla’s stage 6, while normal tooth matched Nolla’s stage 7.^39,40^

Another interesting and rare finding observed in the present case was the mandibular bone altered radiopacity adjacent to the RO teeth. This finding was first reported by Ansari et al^19^ in a 9-year-old nonsyndromic girl who presented a “ghostly” appearance in the right mandible on panoramic radiography; thus, this case is the second one regarding adjacent RO teeth-related sclerotic bone with coarse and irregular trabeculae. Among syndromes featuring RO, there has been published only one case revealing a mandibular bone pattern abnormality to date, which was published by some of the present authors.^8^ Damasceno et al^8^ showed an altered maxillomandibular trabecular pattern associated with a generalized odontodysplasia on CBCT of a 5-year-old girl diagnosed with Hallermann-Streiff syndrome. In both situations (syndromic and nonsyn-dromic RO), a combination of some aspects appears to explain the altered radiopacity in areas surrounding the altered teeth: (1) since teeth displaying RO features do not have a normal development, it may be reasonable disturbance on the alveolar ridge formation process followed by an alteration in the alveolar bone density;^19^ (2) an abnormal periosteal appositional bone formation or a defective endosteal resorption could alter the mandibular bone trabeculae organization.^41^ In a clinical point of view, this radiographically altered bone should be carefully analyzed when planning osseointegrated implants placement.^17^

In order to provide new additional information to the current literature regarding RO and its possible effect on the mandibular bone pattern, it was performed a comparative fractal analysis in the present case. Fractal analysis to evaluate bone microarchitecture has been conducted in panoramic radiograph studies. Presently, fractal dimension was calculated by the box-counting method as published by White and Rudolph.^22^ It has been demonstrated that this methodology is able to show differences in bone density on panoramic radiographs of patients with osteoporosis,^42,43^ hyperparathyroidism,^44^ sickle cell anemia,^45^ chronic kidney disease,^46^ and osteogenesis imperfecta.^47^ Apparently, it is the first study using fractal analysis for characterization purposes in a mandibular case of OR. In this patient, a reduced fractal dimension value was obtained in the trabecular bone area affected by RO when compared with the corresponding nonaffected counter lateral mandibular region. This finding reflects an apparent trabecular bone scarcity, which agrees with Ansari et al^19^ who described the opaque appearance on the panoramic radiograph as a lower density of the alveolar bone around the affected teeth.

In brief, this case describes a rare developmental teeth-related pathology in a boy who showed unusual features on imaging exams, including mandibular involvement and missing permanent teeth. Cone beam computed tomography provided the observation of RO tooth-related GTs, probably a new finding added to the international literature regarding RO, as well as the pulp chamber volume characterization of an affected tooth not published yet. In addition, it was observed an altered fractal pattern of the mandibular bone adjacent to RO teeth, which has not been described to date.

## CONCLUSION

The case is unusual, in that the RO occurred in the mandible. Findings in CBCT and panoramic radiograph revealed peculiarities not yet reported in articles. Therefore, it is important to identify and describe these imaging characteristics, in order to add new information to the current international literature.

## CLINICAL SIGNIFICANCE

This case describes a rare developmental teeth-related pathology in a boy who showed unusual features on imaging exams. The CBCT provided the observation of RO tooth-related GTs, probably a new finding added to the international literature regarding RO, as well as the pulp chamber volume characterization of an affected tooth not published yet. In addition, it was observed an altered fractal pattern of the mandibular bone adjacent to RO teeth, which has not been described to date.
